# Alzheimer’s Disease: Mechanism and Approach to Cell Therapy

**DOI:** 10.3390/ijms161125961

**Published:** 2015-11-04

**Authors:** Takashi Amemori, Pavla Jendelova, Jiri Ruzicka, Lucia Machova Urdzikova, Eva Sykova

**Affiliations:** 1 Department of Neuroscience, Institute of Experimental Medicine, Academy of Sciences of the Czech Republic, Videnska 1083, 142 20 Prague 4, Czech Republic; jendel@biomed.cas.cz (P.J.); j.ruzicka@biomed.cas.cz (J.R.); urdzikl@biomed.cas.cz (L.M.U.); sykova@biomed.cas.cz (E.S.); 2 Department of Neuroscience, 2nd Faculty of Medicine, Charles University, V Uvalu 84, 150 06 Prague 5, Czech Republic

**Keywords:** Alzheimer’s disease, amyloid-β, Tau, mesenchymal stem cells, neural stem cells

## Abstract

Alzheimer’s disease (AD) is the most common form of dementia. The risk of AD increases with age. Although two of the main pathological features of AD, amyloid plaques and neurofibrillary tangles, were already recognized by Alois Alzheimer at the beginning of the 20th century, the pathogenesis of the disease remains unsettled. Therapeutic approaches targeting plaques or tangles have not yet resulted in satisfactory improvements in AD treatment. This may, in part, be due to early-onset and late-onset AD pathogenesis being underpinned by different mechanisms. Most animal models of AD are generated from gene mutations involved in early onset familial AD, accounting for only 1% of all cases, which may consequently complicate our understanding of AD mechanisms. In this article, the authors discuss the pathogenesis of AD according to the two main neuropathologies, including senescence-related mechanisms and possible treatments using stem cells, namely mesenchymal and neural stem cells.

## 1. Introduction

The first case of Alzheimer’s disease (AD) was observed by Alois Alzheimer in 1901, with the histological findings, including “plaques” and “tangles” in the upper cortical layer, published in 1907 [1]. Oskar Fischer also found and described neurite plaques in senile dementia cases in the same year [2]. Fischer’s name had almost vanished from the history of AD until his contributing works were recounted and recognized by Michel Goedert in 2009 [3] and at the 9th International Conference on Alzheimer’s and Parkinson’s diseases held in Prague the same year. Alzheimer’s works, including his clinical notes and brain slides, were rediscovered by Maurer, Volk and Gerbaldo in 1995 (published in 1997) [4], and by Graeber and his group in 1992 and 1997 (published in 1997 and 1998) [5,6], respectively. Alzheimer’s first AD patient was Auguste Deter, a 51 year old female. Rediscovered histological sections have revealed her genetic background; she had a ε3/ε3 Apolipoprotein E (APOE) genotype [6] and a presenilin 1 mutation [7]. However, the latter finding has not been supported by subsequent study [8].

Alzheimer’s disease begins with memory loss of recent events (short-term memory impairment) and finally robs the patients of their sense of self. AD is involved in 50%–70% of dementia cases, and nearly half of people over the age of 85 suffer from it [9,10]. The disease poses a great threat to older individuals and their families, becoming a serious social problem with increasing longevity. AD is characterized by two main pathological findings in the brain: Senile plaques (SPs) and neurofibrillary tangles (NFTs). The former are extracellular aggregates composed of amyloid β (Aβ) peptides, while the latter are intracellular aggregates composed of hyperphosphorylated Tau protein.

In this review, we first describe recent findings concerning any genetic involvement in AD pathogenesis. Following this, our current knowledge of SPs and NFTs in AD pathogenesis is described together with immunotherapeutic efforts. To further understand the causal mechanisms of SPs and NFTs, metabolic changes accompanying advancing age and during AD development are considered, focusing on glial involvement in AD development. For the consideration of future research, there are a few words of caution concerning the use of animal models of AD, including their differences compared to human AD patients. Finally, stem cells in AD brains and their therapeutic potential are discussed.

## 2. Gene Mutations Related to Early-Onset and Late-Onset AD

Early-onset AD (EOAD), defined as occurring before 65 years of age, accounts for less than 10% of AD cases. EOAD with a family link is referred to as familial AD (FAD), most cases of which are linked to autosomal dominant inherited gene mutations: Amyloid precursor protein (APP) (16% of FAD), presenilin 1 (PSEN1) (30%–70% of FAD) and presenilin 2 (PSEN2) (less than 5% of FAD) [11]. AD inherited with these genes is defined as autosomal-dominant AD (ADAD) [12]. Such autosomal dominant AD accounts for approximately 1% of all AD cases. Mutation of the APP gene facilitates Aβ production whilst that of PSEN 1 and PSEN2 increases the production of Aβ_42_ via γ-secretase [13,14].

Late-onset AD (LOAD) occurs after 65 years of age and is also known as sporadic AD (SAD), accounting for 85%–95% of AD cases [15]. The APOE gene is the largest known genetic risk factor for SAD. APOE is the product of a single gene on chromosome 19 [16], is mainly produced by astrocytes and microglia in the brain, and is involved in the transportation and metabolism of cholesterol and triglycerides [17,18]. Three APOE isoforms (APOE2, APOE3, APOE4) with the following population prevalences have been identified as contributing to the disease: APOE3 (77%–78%) > APOE4 (14%–16%) > APOE2 (7%–8%) [19]. The APOE gene exists as three different alleles in humans (ε2, ε3 and ε4). The ε4 allele of APOE is recognized as a major risk factor for SAD, increasing the risk of developing the disease by three-fold in heterozygotes and by 15-fold in homozygotes. [20,21]; however, in sporadic cases its estimated prevalence risk is only 10%–20% [22]. A large scale meta-analysis was performed using a genome-wide association study (GWAS), which revealed 22 associated genetic loci linked to SAD [23,24,25], Detailed descriptions of these genes have been published elsewhere [26,27,28,29,30,31,32,33,34,35,36,37].

SAD is the most common form of AD. In addition to APOE, dozens of other genetic risk factors for SAD have been identified, although further evidence is required to evaluate newly identified risk factors in terms of their functional roles and contributions. Cholesterol metabolism and immune response have been indicated as the primary causes of SAD among many categories used in one analysis [38]. TREM2, CD33 and CR1 are related to the microglial phagocytosis of Aβ [28,31,32]. These additional genetic findings may offer a key to understanding the sophisticated pathological mechanisms of AD, giving us an opportunity to create a suitable animal model of SAD.

## 3. Amyloid Plaques and Immunotherapy

Amyloid precursor protein (APP) appears to play an important role in neural development and neurogenesis. It is cleaved by β-secretase (BACE1) at the N-terminal of an Aβ sequence to form a 99 amino acid fragment C99, which is subsequently cleaved by γ-secretase producing an Aβ fragment and APP intracellular domain (AICD) [39]. This process produces Aβ consisting of 36 to 43 amino acids; Aβ_40_ is the most abundant species (90% of the total Aβ peptide) in normal and AD brains followed by Aβ_42_ [40]. An extracellular fragment of APP binds death receptor 6 (DR6) or p75NTR (DR6 has a much higher affinity for APP than does p75NTR) and triggers the degeneration of cell bodies [41].

Most research is directed at two particular targets: Amyloid accumulation and tangle formation. The former is targeted according to the amyloid cascade hypothesis [42,43,44,45], which is based on the deposition of Aβ protein, the main component of the plaques that drive AD, leading to NFTs, neuronal loss, vascular damage, and dementia [44]. However, amyloid plaque burden poorly correlates with disease severity [46]. On the other hand, elevated levels of Aβ_40_ and Aβ_42_ correspond to the degree of cognitive decline when a single formic acid extract is used [47], suggesting that soluble Aβs, such as amyloid oligomers, correlate with disease severity [48]. Amyloid oligomers have been shown to impair long-term potentiation (LTP) and cognitive function, and the synaptotoxicity of amyloid oligomers has been suggested [49,50]. However, careful analysis is required to examine oligomeric toxicity and to compare data obtained from different laboratories because the ubiquitous protein fractionation technique SDS-PAGE is not a reliable method for analyzing amyloid oligomers. SDS may artificially induce Aβ aggregation and conformational changes [51]. Memory loss at the early stage of AD may be partly due to the synaptic dysfunction induced by amyloid oligomers which cause perturbations in insulin signaling [52,53]. The binding of Aβ oligomers to the cellular prion protein (PrP^C^) activates Fyn, resulting in the disruption of synaptic plasticity [54,55]. Aβ dimers isolated from AD brains induce Tau phosphorylation and NFTs [56]. Aβ oligomers bind to Fz receptors, resulting in the inhibition of Wnt signaling, which in turn causes Tau phosphorylation and neurofibrillary tangles [57]. Aβ induces oxidative stress, endoplasmic reticulum (ER) stress, calcium stress and Tau phosphorylation, and sensitizes neurons to excitotoxicity [58]. Although these findings underpin the amyloid cascade hypothesis, it nevertheless only accounts for less than 1% of AD cases. Importantly, data supporting the amyloid cascade hypothesis come mainly from studies using animal models of ADAD.

Active immunization has been used to treat AD, by targeting Aβ. The trial was halted by the development of aseptic meningoencephalitis, which occurred in 6% of patients and was caused by a T-cell-mediated autoimmune response. Aβ was cleared from the neocortex, but neither cognitive improvement nor changes in Tau pathology, cerebral amyloid angiopathy, or Aβ oligomers were observed [59]. In order to prevent the side effects induced by active immunization, passive immunization was utilized. There were no significant clinical improvements in Phase 1 and 2 studies using a single dose of solanezumab, an IgG1 antiamyloid monoclonal antibody that binds to soluble monomers and lower-molecular-weight Aβ oligomer species, but not to fibrillary Aβ species or higher-molecular-weight Aβ oligomer species [60,61]. Repeated treatment with solanezumab did not show a significant benefit in data obtained from patients with mild or moderate AD dementia, but a slowing of cognitive decline was found in approximately 34% of mild AD patients, diagnosed as ADAS-Cog_14_ (AD Assessment Scale Cognitive subscale) [60,62,63], supporting the suggestion that amyloid-targeted therapy could be more effective when applied at earlier stages of AD or before visible symptoms appear [64,65]. Specific immunization of the neurotoxic Aβ oligomer might be beneficial to circumvent inhibitory damage to the protective physiological benefits of Aβ. Further on-going studies should reveal the efficacy of these antibodies in the treatment of AD patients. Aβ immunotherapies currently used in clinical trials have been described in detail by Goure and colleagues (2014) [61].

## 4. Tau Pathology and Immunotherapy

Tau is a microtubule-associated protein (MAP) required for stabilizing microtubules and neurite outgrowth [66,67]. Normal Tau interacts with tubulin, facilitates its assembly into microtubules and stabilizes their structure [66]. Tau-based neurofibrillary pathology is found in more than 20 neurodegenerative diseases [68]. Phosphorylation of Tau within the microtubule binding repeats (R) is necessary for appropriate neurite outgrowth. The ratio of 3R and 4R Tau isoforms is generally 1:1 in the adult brain, but deviations from this ratio may cause Tauopathies (Tau pathologies) [69].

Hyperphosphorylated Tau spontaneously aggregates into paired helical filaments (PHF), which can subsequently form NFTs. In AD, hyperphosphorylated Tau accumulates, prompting its dissociation from microtubules, thus leading to their destabilization and the disruption of neuronal transport [70]. The number of NFTs correlates with the extent of disease progression in AD but does not correspond to neuronal loss, since memory deficits and neuronal loss precede the formation of NFTs [71]. Tau oligomers, rather than fibrillar aggregates, may be cytotoxic [72]. One study found that learning and memory deficits were exacerbated with increasing Tau oligomers in AD [73]. Synaptic loss and microglial activation precede the onset of NFT formation, reflecting the impaired axonal transport that occurs as a result of Tau hyperphosphorylation [74,75]. Tau pathology is always present in the entorhinal cortex of all people over 75 years of age [76]. The MAP Tau gene itself has been found in different diseases with different forms of dementia other than AD and has been reportedly located on human chromosome 17q21 in frontotemporal dementia with parkinsonism [77], subsequently referred to as frontotemporal dementia and parkinsonism linked to chromosome 17 (FTDP-17). This mutation of Tau induces NFTs composed of hyperphosphorylated Tau protein. Forty four pathogenic MAP Tau mutations have been described in over 100 families [78].

In SAD, Tau-related pathologies are not believed to be downstream of Aβ pathologies, but rather amyloid and Tau pathologies may have dual independent pathways [79]. Phosphorylated Tau is initiated in the brainstem, in particular in the locus coeruleus, followed by the medial temporal lobe, limbic structures, association cortex, and the primary cortices. Conversely, Aβ deposition occurs first in the association cortex and thereafter develops to the lower cortical areas, deep gray matter, brainstem, and cerebellum [80]. It is likely that tangle formation occurs independently of the presence of Aβ. This was indicated in one study by the fact that Aβ vaccination almost entirely cleared Aβ, whilst the severe and progressive tangle pathology remained and clinical improvement was not achieved [81]. This finding encourages the development of AD treatments targeting Tau pathologies. Active immunization using Tau epitopes has been performed to block or reduce Tau pathology, but it also carries the risk of encephalitis or neuronal apoptosis [82]. Passive immunization trials have shown that Tau related pathology could be reduced when the antibody was administered at early time points prior to the onset of Tau pathology [83,84]. Passive immunization with anti-Tau antibodies can reduce Tau pathology and delay the development of motor deficits in P301S transgenic mice [84]; such clinical trials are ongoing.

Therapeutic approaches to prevent Aβ accumulation and Tau hyperphosphorylation should not adversely affect their normal protective physiological functions. Low doses of Aβ have been found to enhance LTP and hippocampal acetylcholine production, resulting in memory improvement [85], whilst APP knockout mice have demonstrated functional impairment, having defects in Ca^2+^-handling, synaptic plasticity and/or neuronal network formation rather than gross structural changes [86]. Tau knockout mice are likely to promote the progression of motor dysfunction with advancing age [87].

## 5. Metabolic Changes in Senescence and AD

### 5.1. Protein Metabolism in AD

A functional decline in protein homeostasis (proteostasis) causes an accumulation of damaged and misfolded proteins in aging cells and diseases such as AD [88]. The endoplasmic reticulum (ER) is the major site of protein synthesis. Unfolded or misfolded proteins accumulate in the ER lumen leading to ER stress, which triggers a complex network of signaling events and cellular processes, known as the ubiquitin-proteasome system (UPS), which relieves stress and re-establishes homeostasis [89]. UPS involves translational arrest, ER-chaperone induction and ER-associated degradation (ERAD). ERAD can remove unfolded proteins through retrograde transport from the ER to the cytosol [90]. If the protective mechanism of the UPS fails to recover homeostasis, pro-apoptotic signals cause the death of irreversibly damaged cells, with excessive and prolonged ER stress resulting in apoptotic cell death. An accumulation of unfolded proteins triggers the dissociation of 78 kDa glucose-regulated protein (GRP78) from the major effectors of the UPS, including inositol requiring enzyme 1α (IRE1α), protein kinase RNA-like ER kinase (PERK) and activating transcription factor 6 (ATF6). PERK and IRE1α are activated by dimerization followed by autophosphorylation. ATF6 translocates to the Golgi apparatus and is cleaved by two proteases, S1P and S2P, to release an active cytosolic fragment (ATF6f) that regulates a subset of UPS target genes involved in ERAD. PERK can phosphorylate α subunits of eukaryotic initiation factor 2 (eIF2α), which arrest protein synthesis and alleviate the overload of proteins inside the ER [91]. When stress cannot be alleviated, ATF4 promotes cell death by upregulating transcription factor C/EBP homologous protein (CHOP) through BH3-only members of the Bcl-2 family. CHOP induces endoplasmic reticulum oxidoreductin-1α (ERO1α) which activates the inositol trisphosphate receptor (IP3R) stimulating calcium release from the ER, and leading to calcium overload and apoptosis by mitochondrial uptake. Increased ERO1α induces hyperoxidation in the ER that may promote cell death [92]. Activated IRE1α can bind tumor necrosis factor (TNF) receptor associated factor 2 (TRAF2), which in turn stimulates apoptosis signal-regulating kinase 1 (ASK1) and leads to the activation of c-Jun N-terminal kinase (JNK) and p38 mitogen-activated protein kinase (p38 MAPK), consequently inducing autophagy and apoptotic cell death [93,94]. JNK and p38 MAPK are also involved in Tau phosphorylation [95,96]. Chaperone BiP, PERK and eIF2α decrease during aging [97]. ER stress induces inflammation via the activation of NF-κB [98], which can activate BACE1 resulting in amyloidogenesis [99]. ER stress can also activate Tau kinase, glycogen synthase kinase 3β (GSK-3β), which enhances NFT formation [100].

The UPS and autophagy systems are indispensable for the maintenance of proteostasis as misfolded and damaged proteins must be efficiently refolded or removed. Chaperones play a key role in the proteostasis system and in sensing misfolded proteins, which are directed to the protein degradation pathways when refolding fails [101]. Almost all aging organisms show a gradual decrease in UPS and autophagy activity [102]. Among the heat shock proteins (HSPs), known as molecular chaperones, HSP90, HSP70, and HSP32, which are increased in the AD brain, induce the production of IL-6 and TNFα and increase the microglial phagocytosis and clearance of Aβ_42_ by NF-κB and p38 MAPK activation, via Toll-like receptor 4 (TLR4) [103]. HSP22 and HSP27 bind to fibrillar amyloid plaques to inhibit further fibrillarization [101]. Proinflammatory cytokines such as IL-1 and TNF-α facilitate the phosphorylation of small heat shock proteins [104]. GRP78, also known as binding immunoglobulin protein (BiP), is a member of the HSP70 protein family, which regulates APP and Aβ secretion by modulating the interaction between APP, β-secretase and γ-secretase. GRP78 is required for stress-induced autophagy and plays a central role in regulating UPS [105].

For stabilization, Tau first binds to the co-chaperone heat-shock cognate protein-70 (HSC70), but if this does not occur, it binds to HSP70 for degradation [106]. Tau can be degraded via the ubiquitin-proteasome and lysosomal pathways. The C terminus of HSP70-interacting protein (CHIP) is the ubiquitin ligase of Tau. Reduced CHIP levels increase the accumulation of Tau aggregates in transgenic mice and are present in AD brains [107]. HSP27, HSP70 and CHIP can recognize abnormal Tau and reduce its concentration by facilitating its degradation and dephosphorylation [104]. Akt, referred to as protein kinase B (PKB), can hyperphosphorylate Tau directly or indirectly through GSK-3β and PARK1/PARK2, preventing CHIP-induced Tau ubiquitination, and is present in AD at elevated concentrations [108].

### 5.2. Cholesterol Metabolism (Lipid Rafts and PrP^C^) in AD

The human brain contains about 25% of the body’s total cholesterol [109]. Since the blood brain barrier (BBB) prevents the uptake of lipoproteins, brain cholesterol must be derived from *de novo* synthesis [110]. Alterations in the distribution of lipids within brain cell membranes during aging are considered a risk factor for AD [111]. Ganglionsides, especially GM1, bind with Aβ and convert soluble nontoxic Aβ into aggregated toxic Aβ, *i.e.*, the conformational transition from α-helix to β-sheet; this step is considered to be critical in AD development [112]. An increase in cholesterol concentration in neuronal membranes accelerates Aβ binding to GM1 (GAβ), which subsequently promotes Aβ fibrillation [113,114]. GAβ-induced amyloidogenesis was suppressed by pretreatment with a sphingomyelin synthase inhibitor. Sphigomyelin is also involved in GAβ generation [115]. Cholesterol, sphingomyelin, and GM1 are all contained in plasma membrane microdomains known as lipid rafts and are abundant in cholesterol and sphingolipids, serving as a platform for cellular signaling as well as protein-lipid and protein-protein interactions [116,117]. APP, BACE1, the γ-secretase subunits and Aβ are found in raft domains [118]. Increased cholesterol levels upregulate Aβ formation, whereas low cholesterol levels relocate the major α-secretase, ADAM10, from raft domains to non-raft regions of the membrane, resulting in increased non-amyloidogenic processes [119,120]. In contrast, the movement of BACE1 from non-raft to raft domains causes an upregulation of soluble β-cleaved APP and Aβ production. Cholesterol binds to C99, which promotes amyloidogenic processing and, in turn, causes alterations in cholesterol homeostasis in the Golgi and plasma membrane [121].

APP intracellular domain (AICD) released from APP by PS1-dependent γ-secretase activity regulates plasmalogen synthesis [122,123]. Reduced plasmalogen levels in the AD brain oppose the inhibitory activity of γ-secretase, resulting in increased Aβ production. AICD also regulates sphingolipid synthesis via serine palmitoyltransferase and may control the composition of lipid rafts and APP processing [124]. Lipid rafts are components of cell membranes that integrate signaling pathways and regulate physiological cellular function [121]. Lipid destabilization in lipid rafts occurs as an early event in the pathogenesis of AD from the frontal and entorhinal cortices, and may result in the amyloidogenic processing of APP [125]. Membrane ceramides, the major component of lipid rafts facilitate the trafficking of BACE1and γ-secretase to lipid rafts leading to Aβ production [126]. β-Secretase and γ-secretase are located in cholesterol-rich lipid rafts, while the non-amyloidogenic α-secretase is associated with the membrane surface, outside the raft domains. β-Secretase activity is increased by cholesterol [16]. The amyloid-degrading enzymes neprylisin (NEP) and insulin-degrading enzyme (IDE) are also associated with lipid rafts [127,128,129], suggesting that lipid rafts may be involved in Aβ degradation.

The cellular prion protein (PrP^C^) is a normal protein found on cell membranes. It is neuroprotective and plays important roles in defending against oxidative stress and maintaining metal ion homeostasis in the brain [130]. In contrast, in AD, Aβ oligomers binding to PrP^C^ interrupt the protein’s inhibitory effects on BACE1 resulting in increased Aβ production. The binding of Aβ oligomers to PrP^C^ activates Fyn, which is a member of the Src family of tyrosine kinases and regulates the internalization and synaptic localization of NR2B-containing NMDAR [131]. Fyn activation induced by Aβ oligomer-PrP^C^ complexes drives tyrosine phosphorylation of the NR2B subunit of NMDARs, which is also localized in lipid rafts [120]. NMDAR phosphorylation in turn causes LTP inhibition, oxidative stress, apoptosis and calcium dysregulation, resulting in neuronal loss and memory impairment [51,132,133]. Aβ oligomer-mediated early synaptic dysfunction depends on the phosphorylation of NMDAR subunits [134]. PrP^C^ and Fyn are located at synapses and enrich the postsynaptic density (PSD). However, PrP^C^ is localized on the outer surface of the membrane where it attaches to the lipid bilayer via a glycosylphosphatidylinositol (GPI) anchor, whereas Fyn is present on the inner side of the membrane. Lipid rafts provide the opportunity for the interaction of PrP^C^ and Fyn [135]. Age- and disease-dependent disruption of lipid rafts may result in the inability of PrP^C^ to control BACE1 [124]. Furthermore, since lipid rafts are strongly concentrated in hippocampal neurons, the interaction of Aβ oligomer and PrP^C^ may induce memory deficits [136]. The Aβ oligomer–PrP^C^–Fyn pathway seems to link to synaptic loss and memory impairment, the most prominent aspects of AD. On the other hand, some studies have cast doubt on the involvement of PrP^C^ in memory impairment. Ablation or overexpression of PrP^C^ had no effect on hippocampal synaptic plasticity and oligomer-induced cognitive impairment [137,138]. Recent studies suggest that these conflicting results may be attributed to differences in soluble Aβ, the location of its binding site in PrP^C^ and/or the animal models used [139,140]. Among soluble Aβ, protofibrils have a high affinity interaction with PrP^C^. Treatment with an antibody that binds PrP^C^_93–109_ prevents neuronal cell death by Aβ oligomers, but antibodies that bind PrP^C^_144–152_ or PrP^C^_213–230_ fail to block Aβ-induced neurotoxicity. Tau [128] and proline-directed serine/threonine kinases, such as cyclin-dependent kinase 5 (Cdk5) [141] and GSK-3β [142] that are recognized as prime mediators in the hyperphosphorylation of Tau, have been detected in lipid rafts. It is possible that lipid rafts may serve as domains between Tau and its related kinases. Cdk5 is activated in neurons by the neuron-specific activator p35 and is involved in brain development and synaptic activity under normal physiological conditions [143]. In AD, various stressors such as ischemia, oxidative stress, mitochondrial dysfunction, neuroexcitotoxicity, Aβ exposure, calcium imbalance, and inflammation lead to the elevated influx of calcium into the cytoplasm, which in turn activates the calpain-mediated cleavage of p35 to p25 [144]. The half-life of p25 is longer than that of p35. Through its p10 myristoylated N-terminal end, p35 is bound to the membrane, while in contrast p25 localizes to the cell soma because of its lack of p10 [145]. These differences form a more stable and hyperactive Cdk5/p25 complex, which causes aberrant hyperphosphorylation of Tau, leading to neurodegeneration and cell death. Calpain activation leading to p25 accumulation and elevated Cdk5 activity has been found in the AD brain [146]. Fyn activates GSK3β and Cdk5 and can also hyperphosphorylate Tau at tyrosine 18 by itself. This tyrosine phosphorylated Tau has been found in NFTs in the AD brain [147,148]. Tau binds and sequesters Fyn to alter its localization in the neuron. This altered Fyn localization may in turn activate Fyn via Aβ [149]. Thus, Tau can interact with Fyn in dendrites, which stabilizes the interaction of NMDAR with the postsynaptic density (PSD) protein PSD-95 and mediates Aβ-induced-neurotoxicity [150].

### 5.3. Glucose Metabolism in AD

Up to 50% of the body’s total glucose is consumed in the brain. However, this consumption of glucose decreases with age and in AD [151]. Glucose deprivation is used as an energy deficiency for *in vitro* induced eIF2α phosphorylation, which increases BACE1 levels and thereby promotes amyloidogenesis in AD [152,153]. Glucose transporters (GLUT) 1 and 3 play an important role in transporting glucose to neurons [154]. Levels of GLUT1 and GLUT3 decline in AD, which results in decreased uridine diphosphate *N*-acetylglucosamine (UDP-GlcNAc) production derived from glucose via the hexosamine biosynthesis pathway (HBP) [151]. Protein *O*-GlcNAcylation is a post-translational modification that includes the attachment and removal of *O*-linked β-*N*-acetylglucosamine (*O*-GlcNAc) to/from serine and threonine residues of nuclear and cytoplasmic proteins; these processes are regulated by *O*-GlcNAc transferase (OGT) and *O*-GlcNAcase (OGA), respectively [155]. OGT and OGA are abundantly distributed in the brain, especially in the hippocampus [156]. Tau phosphorylation is inversely regulated by *O*-GlcNAcylation [157]. Downregulation of protein phosphatase-2A (PP2A), which regulates the activity of several Tau kinases and impairs brain glucose metabolism, contributes to abnormal hyperphosphorylation of Tau in AD [158]. In AD brains, the level of *O*-GlcNAcylation was 22% lower compared to controls [159]. *O*-GlcNAcylation and PP2A regulate Tau phosphorylation at overlapping though partially different phosphorylation sites [151]. Impaired glucose metabolism leads to decreased Tau *O*-GlcNAcylation and causes abnormal hyperphosphorylation of Tau, resulting in the NFTs observed in AD. Furthermore, *O*-GlcNAcylation influences the APP processing, which results in increased non-amyloidogenic processing by facilitating α-secretase; with increasing neuroprotective α-secretase cleaved from soluble APP fragments, Aβ secretion declines [155]. OGA and OGT in synaptosomes regulate *O*-GlcNAcylation of synaptic proteins. The inhibition of OGA causes increased *O*-GlcNAcylation of pre-synaptic proteins and enhances LTP, which is related to memory function [160].

### 5.4. Oxidative Stress and Metabolism

Oxidative stress is caused by an imbalance between pro-oxidant and antioxidant systems and is exacerbated during aging and AD. An accumulation of reactive oxygen species (ROS), which is particularly characteristic of oxidative stress, is mainly produced by mitochondria and causes damage to lipids, cellular proteins, nucleic acids and glucose. The consequences of such damage are seen as lipid peroxidation, protein oxidation, DNA/RNA oxidation, and glycoxidation [161]. Glutathione is the most prevalent antioxidant in the brain and plays a role in the detoxification of ROS [162]. Levels of glutathione decrease with age [163] and in AD [164]. Decreased intracellular glutathione leads to the release of pro-inflammatory factors TNF-α, IL-6 and nitrite ions, and the activation of P38 MAPK, JNK and NF-κB in microglia and astrocytes [165]. JNK-dependent activation of γ-secretase is promoted by hydrogen peroxide (H_2_O_2,_ a source of ROSs), resulting in Aβ production [166]. Manganese superoxide dismutase (MnSOD) is an antioxidant enzyme that protects mitochondria from oxidative stress. Its inactivation has been observed in an animal model of AD, resulting in the promotion of mitochondrial dysfunction [167]. High concentrations of Cu, Zn and Fe have been found around amyloid plaque [168]. Since Aβ is a metalloprotein that can bind Cu, Zn and Fe ions [169], this might reflect an accumulation of such metals in the AD brain. Complexes of Aβ and Cu/Fe can generate ROS such as H_2_O_2,_ leading to Aβ toxicity [170]. In particular, the Aβ/Cu complex catalyzes tyrosine oxidation by H_2_O_2_ leading to dityrosine crosslinking of Aβ that contributes to the stabilization of oligomeric species and amyloid fibrils [171]. Levels of dityrosine were found to be elevated in the hippocampus and neocortical regions of the AD brain [172]. In contrast, Zn seems to rescue cells from toxic conditions by reducing the Cu-dependent formation of H_2_O_2_ [173]. However, the dyshomeostasis of Zn induced by Aβ leads to microtubule destabilization and increased Tau phosphorylation [174]. Thus, Aβ can act as both an antioxidant and also a pro-oxidant according to its redox properties. Advanced glycation endproducts (AGEs) are formed by non-enzymatic glucoxidation. The receptor for AGE (RAGE) can bind Aβ as well as AGEs. During AD progression, the expression of RAGE is upregulated in microglia, neurons and endothelial cells surrounding senile plaques [175]. The binding of AGEs and Aβ to RAGE activates NF-κB, which in turn induces the release of various cytokines such as IL-1, IL-6, and TNF-α [176]. This binding also fosters ROS generation by activating NADPH oxidase (NOX), resulting in AD progression [177]. Levels of RAGE, AGEs and Aβ increase in the hippocampus of AD patients, including the dentate gyrus (DG) and CA3 pyramidal neurons. This finding corresponds with the short-term memory loss in AD patients caused by neuronal dysfunction in the hippocampus [178]. The binding of RAGE with AGEs or Aβ activates BACE 1, resulting in Aβ production [179]. Aβ and AGEs can induce mitochondrial dysfunction leading to neurodegeneration [180]. RAGE is also localized in the BBB and mediates the influx of Aβ into the hippocampus and cortex across the BBB [181,182]. AGEs are likely to foster amyloidosis by forming protease-resistant peptides and proteins, leading to protein deposition, and NFT formation by the glycation of Tau, which may stabilize PHF aggregation [177]. Oxidative stress-mediated JNK activation and decreased Wnt signaling followed by GSK-3 activation are required for the development of AD. Both are connected to the forkhead-box O (FoxO) response, which is critically involved in the upregulation of antioxidative pathways and apoptosis [183]. Lipid peroxidation induced by Aβ oligomers in the lipid layer fosters lipid peroxidation products including 4-hydroxy-2-nonenal (HNE), malondialdehyde, F2-isoprostanes, and 2-propyn-1-ol [184]. Among these, HNE has been shown to accelerate the formation of Aβ oligomers and protofibrils; this process in turn leads to lipid peroxidation, which produces more HNE and Aβ oligomers [185]. Increased levels of HNE have been observed in the hippocampus of AD patients [186].

### 5.5. Insulin Metabolism and AD

Recently, accumulating evidence has cast a spotlight on type 2 diabetes mellitus as a potent risk factor for AD development, which is likely to be mediated by insulin and insulin-like growth factors (IGF-1, IGF-2). Insulin receptors (IRs) are distributed over the brain, with high levels detected in the olfactory bulb, cerebral cortex, hippocampus, hypothalamus, and cerebellum [187]. In contrast, IGF-1 receptors (IGF-1Rs) are highly expressed in the cerebral cortex, hippocampus, and thalamus [188,189]. Signaling via these receptors exerts an effect on both neuronal and glial functions, including glucose metabolism and energy homeostasis [190]. Insulin receptor substrates (IRS) are critical in insulin signaling and contribute to the maintenance of cell growth, cell survival, and cellular metabolism [191]. There are four members: IRS-1, IRS-2, IRS-3 and IRS-4 [192]. IRS-1 and IRS-2 are the main mediators of the IR/IGF signaling pathway [193]; mice deficient in these substrates showed accelerated Tau hyperphosphorylation [194,195,196]. Similarly, levels of IRs, IGF-1R, IRS-1 and IRS-2 are reduced in AD brains [197], which suggests that reduced insulin and IGF-1 signaling may result in the hyperphosphorylation of Tau by mediating protein phosphatase-2A (PP2A) and glycogen synthase kinase 3β (GSK-3β) [193,196]. Alternatively, this signaling pathway may regulate phosphatidylinositol 3-kinase (PI3K), which in turn activates protein kinase B (PKB) that regulates GSK-3α, which is related to Aβ production and GSK-3β, also known as Tau kinase [198,199]. The impaired signaling pathway may induce the inactivation of PI3K and PKB and disinhibit GSK-3. During aging, similar reductions occur for neuronal glucose metabolism, insulin levels and IR density [200]. Serine phosphorylation of IRSs inhibits insulin signal transduction and contributes to peripheral insulin resistance [201], which is partly mediated by pro-inflammatory cytokines; prolonged resistance is exacerbated by aging and obesity, resulting in glucose intolerance, hyperlipidemia, hypertension, polycystic ovarian syndrome, and type 2 diabetes mellitus [202]. The pro-inflammatory cytokine TNF-α fosters serine phosphorylation of IRS-1 and IRS-2 via JNK binding with IRS proteins, inhibiting subsequent signaling pathways including PI3K/PKB and PI3K/Akt and leading to amyloid deposits and Tau hyperphosphorylation [202,203]. The phosphorylation of serine residues inhibits insulin-stimulated tyrosine phosphorylation [202], which prevents IRSs from binding to IR and IGF receptors and instead directs IRSs towards proteasomal degradation, leading to insulin/IGF resistance [197]. The impairment of insulin/IGF signaling caused by insulin/IGF resistance, characterized by reduced IR and IGF receptor binding to IRSs and a decreased ability to respond to insulin/IGF stimulation, causes oxidative stress, mitochondrial dysfunction, and inflammation. In turn, ROSs produced by oxidative stress and mitochondrial dysfunction as well as pro-inflammatory cytokines secreted during inflammation exacerbate insulin/IGF resistance, which is characteristic of both AD and type 2 diabetes mellitus [200,204,205]. Brain insulin signaling plays an important role in learning and memory [206] and declines with age [207]. Insulin and IGF-1 can protect neurons against Aβ-induced synaptic toxicity [189,208]. Similarly, insulin-degrading enzyme (IDE), also known as insulin protease, can degrade Aβ [209]. IDE is controlled via the insulin-PI3K-Akt signaling pathway, the impairment of which leads to a reduction of IDE [210], which also appears to be involved in Aβ accumulation. The APOE ε4 allele is believed to play an important role in insulin’s effects as AD patients without the APOE ε4 allele showed beneficial effects following memory impairment, whilst those with it had none [211]. Furthermore, IDE in the hippocampus is reduced by approximately 50% in AD patients with the APOE ε4 allele compared to those without it [212]. In light of this, gene expression backgrounds should be taken into account when evaluating the effects of insulin on patients and animal models of AD.

## 6. Glia and AD

Recently, the role of glia in AD pathogenesis has attracted greater interest due to its growing significance. In this section, the AD-related functions of microglia and astrocytes will be described. In the adult human neocortex, the glia/neuron ratios are 1.32 for females and 1.49 for males. Approximately 75% of neocortical glial cells are oligodendrocytes, 20% are astrocytes, and 5% are microglia. The number of neurons and oligodendrocytes decreases between 20 and 90 years of age by 10% and 27%, respectively, but that of astrocytes remains constant [213].

### 6.1. Microglia

Activated microglia are observed in AD, characterized by short, thickened and less ramified processes. In the aged human brain, microglia are de-ramified and characterized by fragmented processes and bulbous swellings. However, these age-related morphological changes have not been observed in the rodent brain [214]. Microglia have been shown to exert both proinflammatory and anti-inflammatory effects. The former is characterized by the secretion of proinflammatory cytokines, including IL-1β, IL-6 and TNF-α, resulting in the impairment of neurogenesis [215,216], while the latter involves the production of GFs such as IGF-1, which stimulates neurogenesis [217]. IL-1β released from microglia also increases Tau phosphorylation through a p38 MAPK pathway [218].

Microglia are regulated by fractalkine and CD200. Fractalkine is a 373 amino acid protein known as chemokine (C–X3–C motif) ligand 1(CX_3_CL1) and is expressed by neurons with particularly high levels seen in hippocampal neurons [219]. It binds to G protein-coupled receptors (CX_3_CR1) mainly expressed by microglia [220] and inhibits the production of IL-1β, TNF-α, IL-6 and inducible NO synthetase (iNOS) in microglia through the PI3K pathway [221,222]. Hippocampal CX3CL1 mRNA expression and CX3CL1 levels significantly decrease with age in correlation with increases in IL-1β concentrations [222]. Thus, CX_3_CL1/CX_3_CR1 interaction seems to play an important role in the release of proinflammatory substances from activated microglia. CX_3_CL1 also protects against excitotoxicity leading to neuronal death through the activation of the ERK1/2 and PI3K/Akt pathways [223,224]. The level of plasma soluble CX_3_CL1 was markedly higher in patients with mild to moderate AD than in those with severe AD [225], and the level of tissue CX_3_CL1 was lower in the hippocampus and the frontal cortex of AD patients [226]. The fractalkine signaling pathway mediates communication between microglia and neurons which is downregulated in AD brains, but further investigation is required to understand the precise mechanism of fractalkine signaling based on the stage of AD.

CD200R is an inhibitory receptor on microglia, which are maintained in a quiescent state by the interaction between CD200R and CD200, a transmembrane glycoprotein expressed on neurons [227]. A deficiency in CD200–CD200R interaction may contribute to chronic inflammation leading to AD progression [228]. There are decreased levels of CD200 in aged rats compared with adults [229] and decreased CD200 mRNA expression in the rat hippocampus accompanying increasing age [230]. A significant decrease of both CD200 and CD200R within the brain, with a specific deficit of CD200R mRNA in the hippocampus and interior temporal gyrus, was observed in AD brains compared with matched non-demented tissue [231]. The activation of TLR2 and TLR4 was exacerbated in CD200-deficient mice and exerted a negative effect on LTP [232]. The interruption of the CD200 and CD200R interaction may induce LTP impairment in the hippocampus leading to dementia.

Microglia are involved in the phagocytosis of Aβ and in the inflammatory responses that play important roles in AD progression, and are also regulated by Fc gamma receptors (FcγRs) and TYRO protein tyrosine kinase-binding protein (TYROBP, also known as DAP12) [233,234,235]. There are two fundamental pathways to clear Aβ from the brain. One is mediated by several receptors that are expressed in microglia, including scavenger receptors (SR), formyl peptide-receptor-like 1 (FPRL1), complement receptors, FcRs, and TREM2 [236]. The second pathway involves processing by Aβ-degrading enzymes such as neprilysin (NEP), insulin-degrading enzyme (IDE), matrix metalloprotease (MMP) and cathespin B [237,238,239,240]. Microglial clearance of Aβ appears to be dependent on age and also on the stage of the disease since Aβ is more effectively removed in the early stages of AD [241]. In addition, beclin 1 is known to regulate the retromer complex, which is required to maintain phagocytic receptor recycling and phagocytosis. Beclin 1 deficiency impairs the recycling of the phagocytic receptors CD36 and TREM2. Furthermore, the levels of beclin 1 and retromer protein are significantly reduced in microglia isolated from human AD brains, which may lead to an insufficient microglial phagocytic capacity to clear Aβ [242]. The inflammasome NLRP3, also known as NALP3 or CIAS1, is involved in the Aβ-induced activation of caspase-1 in microglia which in turn mediates the cleavage of IL-1β and IL-18 precursors, leading to the release of IL-1β and IL-18 [243]. The phagocytic activity of microglia is attenuated by pro-inflammatory cytokines such as IFN-γ, IL-1β, and TNF-α, which likely skew microglia towards the pro-inflammatory M1 phenotype [244]. NLRP3 activation adversely affects the microglial clearance of Aβ, and inhibition of NLRP3 can induce microglial phagocytosis and an immunosuppressive M2 phenotype resulting in increased Aβ clearance [245].

### 6.2. Astrocytes

Astrocytes regulate extracellular ionic concentration, water homeostasis and the acid-base balance in the brain, mediate the production and clearance of neurotransmitters, and affect glucose supply, antioxidative defense mechanisms, and synaptic regulation by producing various cytokines, chemokines and growth factors [246,247,248,249]. Anti-oxidants in astrocytes (mainly glutathione and ascorbate) protect the brain against oxidative stress [250]. Pro-inflammatory molecules and cytokines produced and released by activated astrocytes can cause the further activation of astrocytes, thus perpetuating inflammatory signaling cycles, and may lead to Aβ production by activating β- and γ-secretases [251,252]. Aquaporin4 is the most abundant water channel in the brain and is widely expressed in the astrocyte plasma membrane [253]. A failure to promote the circulation of interstitial fluid via astrocytic aquaporins may cause an accumulation of misfolded proteins in AD brains [246].

Glutamate is converted to glutamine by glutamine synthetase (GS) in astrocytes. The glutamine is released and taken up into neurons and converted into glutamate by mitochondria glutaminase. Aβ_42_ and oxidative stress significantly decrease GS activity, especially in the hippocampus and neocortex of the AD brain, resulting in an increase in glutamate levels and prolonged NMDA receptor activation [254]. GLT-1 is oxidatively modified by binding to the lipid peroxidation product HNE. This process is facilitated by excessive Aβ_42_ and leads to the inhibition of glutamate transport and increased extraneuronal glutamate accumulation that consequently results in cell death [255]. AD patients have a significant reduction in glutamate transporter activity, associated with increased excitotoxicity and neurodegeneration [256]. Astrocytes are major players in glutamate uptake in the extracellular space and thus keep extracellular glutamate below toxic levels. TNFα downregulates GLAST/EAAT1 and significantly reduces GS expression, resulting in increased excitotoxicity in neurons *in vitro* [257,258]. An age-dependent decrease in GS-positive astrocytes was reported in the hippocampus of 3xTg-AD mice, and GS expression in astrocytes was reduced in the medial prefrontal cortex of the same transgenic mice by the age of 12 months compared with age-matched controls [259,260]. The region-dependent effect of GS should be taken into account when evaluating glutamate neurotoxicity in AD.

Astrocytes are also involved in the clearance of Aβ as well as being a source of Aβ. Although neurons are the major source of Aβ, microglia and astrocytes appear to produce Aβ peptides [261]. The degradation of Aβ is achieved by NEP, IDE, and MMP [262], which are also expressed by astrocytes [262,263,264].

The majority of apolipoprotein E (APOE) is synthesized by the liver [265], but it is also partly produced by astrocytes [266] and microglia [267] in the brain. APOE has a receptor-binding site in its N-terminal domain and a lipid-binding site in its C-terminal domain [268]. APOE receptors include low-density lipoprotein receptors (LDLR), LDL receptor-related protein 1 (LRP1), very low-density lipoprotein receptors (VLDLR), and APOE receptor 2 (APOER2) [269]. LDLR and LRP1 are endocytic receptors, whilst VLDL and APOER2 are signaling receptors [270]. LDLR is a cell surface receptor that regulates APOE in the brain and whose gene is the major risk factor for SAD. Deletion of LDLR causes a decrease in Aβ uptake, whereas LDLR overexpression significantly enhances the uptake and clearance of Aβ by astrocytes [271].

Glia are thus deeply involved in metabolic changes and complicated signaling pathways during AD progression. Although DNA damage in the hippocampal astrocytes of AD brains and an increased population of astrocytes from the frontal cortex of aged individuals and AD patients have been reported [272,273], further intensive studies are required to elucidate their causal relationship to AD pathogenesis and development and to use their therapeutic potential as a target for AD treatment and prevention. Once Aβ starts to abnormally accumulate, an inflammatory response and phagocytosis are promoted in microglia and astrocytes in order to clear it. Conversely, persistent inflammation facilitates Aβ production, and phagocytic ability is reduced with age or during the late stage of AD, resulting in Aβ deposits. An age-dependent decline in Aβ clearance and the augmentation of the inflammatory response by glia are also critical for AD pathogenesis.

## 7. Models of AD and Senescence

### 7.1. Animal Models of AD

Most animal models of AD incorporate modifications to three genes related to ADAD (APP, PSEN1 and/or PSEN2). When using these animal models, the following caveats should be kept in mind: (1) cases of ADAD make up less than 1% of human AD cases; (2) the mechanisms of FAD are different from those of SAD; (3) ADAD can be well explained by the amyloid cascade hypothesis, which is based on amyloid deposition leading to tangle formation; in contrast, SPs and NFTs occur independently in different regions of the brain in SAD; (4) synaptic and neuronal loss, the major cause of human AD symptoms, cannot be addressed in most of these animal models. The 5xFAD animal model co-expresses human APP with the Swedish, Florida and London mutations and human PSEN1 with the M146L and L286V mutations and is known to show neuronal loss, but without NFTs [274]. To induce Tau pathology in an animal model, gene mutations discovered in FTDP-17 are used. The triple transgenic mouse model of AD (3xTg-AD) was generated using three transgenes (APP with the Swedish mutations, PSEN1 with M146V mutations, and Tau with P301L mutations). This animal model shows extracellular Aβ deposits in the frontal cortex at 6 months of age, spreading to the hippocampus by 12 months when Tau pathology appears in the hippocampus; however, no neuronal loss is observed [275,276]. Human Aβ can be expressed in AD transgenic mice, but human C1q (complement protein) cannot. The activation of human C1 by human Aβ is more effective than that of mouse C1 [277].

**Figure 1:**
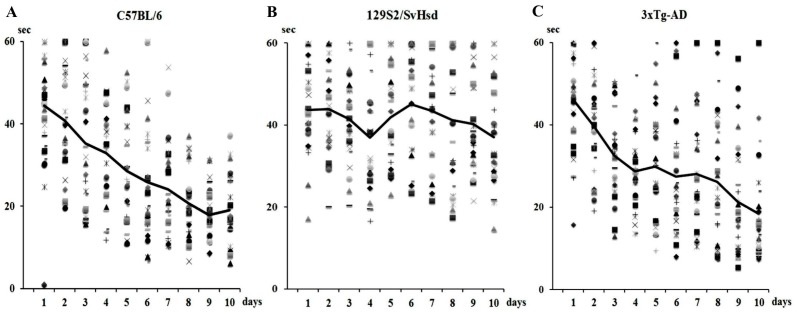
The latency in seconds to find a hidden platform within 60 s over 10 consecutive days of testing is presented for each group: C57BL/6 (**A**); 129S2/SvHsd (**B**); and the triple transgenic mouse model of Alzheimer’s disease (AD) (3xTg-AD) (**C**). Latencies obtained from individual animals are plotted by different marks. Solid black lines show average latencies calculated for each day.

**Figure 2:**
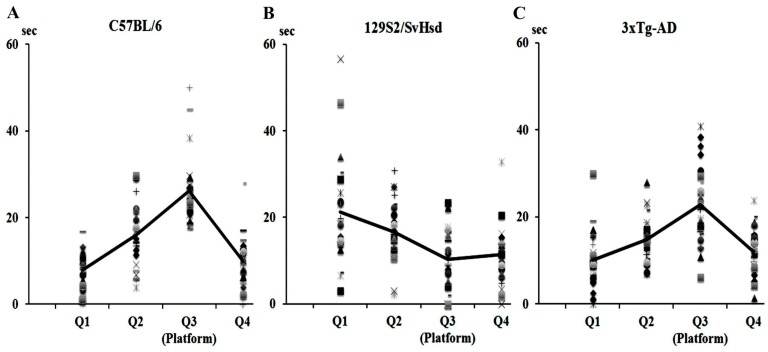
The total time (in seconds) spent in each quadrant (Q1, Q2, Q3, and Q4) during a 60-s probe trial (without the escape platform which was placed in Q3 during the 10-day training) is presented for the three strains of mice (**A**–**C**). Each individual animal’s time is plotted by different marks. Solid black lines show the mean time spent in each quadrant.

Further cautions should be considered in regards to the strains used to prepare the transgenic animal models. For example, 3xTg-AD mice were generated from a hybrid of C57BL/6 mice and F1 animals of 129X1/SvJ and 129S1/Sv. We compared spatial reference memory performance using the Morris water maze (MWM) test (see Supplementary Material) in 3xTg-AD (*n* = 28), C57BL/6 (*n* = 25) and 129S2/SvHsd (*n* = 24) mice, which were used as the 129 substrain. Figure 1 shows the latencies over 10-days training in the MWM test; the mouse, placed in one of four quadrants of the circular pool, had to find a platform hidden 1 cm below the water, made opaque using a non-harmful white color, within one minute. Four trials were given to each animal every day. The results obtained from the individual mice at 3 months of age are indicated by different marks. Over the ten-day training period, all C57BL/6 mice demonstrated decreased latencies for finding the submerged platform, with a final latency of 19.1 ± 1.7 s (mean ± SEM) (Figure 1A). The majority of C57BL/6 mice demonstrated very similar levels of skillfulness, also illustrated by shortened latencies. In contrast, the results observed in 129S2/SvHsd mice highlight how this strain scarcely learned the task at all during the training period, with their average latencies, indicated by a solid black line, not showing any improved performance in finding the platform (Figure 1B). Their final latency was 37.0 ± 2.6 s. 3xTg-AD mice showed a similar improvement to that of the C57BL/6 mice when performing the task, but individual animals had a very wide variation in latencies compared to C57BL/6 mice (Figure 1C). Most of the 3xTg-AD mice could complete the task by decreasing their latency over time, but some of them never learned the task. Their final latency was 18.4 ± 2.3 s. There were also significant differences between C57BL/6 and 129S2/SvHsd mice and between 3xTg-AD and 129S2/SvHsd mice in the last performances (*p* < 0.01). During a probe trial in which the hidden platform was removed, the animals had to place themselves in the quadrant where the platform was previously located within a one-minute time-frame (Figure 2); a stay of less than 15 s was considered to be random chance. All C57BL/6 mice clearly spent the majority of their time in the correct quadrant; the time spent in the correct quadrant (Q3) was 26.2 ± 1.6 s (A); In contrast, most of the 129S2/SvHsd mice did not orient themselves towards the correct quadrant and spent a very short amount of time in the target area (10.3 ± 1.4 s) (B); The transgenic mice spent on average more time in the target quadrant (22.8 ± 1.6 s), but individual animals showed wide differences in the time spent in the target area (C). Statistical differences were found between C57BL/6 and 129S2/SvHsd mice and between 3xTg-Ad and 129S2/SvHsd mice in the probe trial (*p* < 0.01). Varying abilities in task performance of the MWM test have been previously described among the substrains of 129. Some of them, including 129/J, 129/Sc and 129/SvJ, did not show good performance in the MWM test, whilst satisfactory performance was observed in 129/SvEvTac, 129/Ola and 129/Sv [278,279]. Accordingly, to evaluate differences in cognitive ability between mutant and control mice, careful consideration should be given to the genetic differences between the strains used as animal models and control animals [280]. Therefore, it is recommended that large sample sizes be used to compensate for genetic and epigenetic variability [281].

### 7.2. Animal Model of Senescence

An animal model of senescence, senescence accelerated mouse prone 8 (SAMP8), which is a non-genetically modified strain of mice with an accelerated aging process [282,283], displays amyloid plaques, Tau phosphorylation and oxidative stress [284,285] as well as early onset senility and a shortened lifespan. In this animal model, transplantation of whole bone marrow into irradiated mice improved cognitive ability by normalizing proinflammatory cytokines and oxidative markers [286]. Their aging includes oxidative stress, chronic inflammation, calcium dyshomeostasis, chromosomal instability and nuclear and mitochondrial DNA damage [287].

## 8. Stem Cells for Treating and Modeling AD

Although tremendous efforts have been made to delay AD progression as well as ameliorate and cure AD symptoms, only four cholinesterase inhibitors (donepezil, galantamine, reivastigmine, and tacrine, which is rarely prescribed because of its associated side effects, especially liver damage) and an NMDAR antagonist (memantine) have been approved by the U.S. Food and Drug Administration for AD treatment. However, these drugs are not designed to halt or reverse the underlying process of AD, but rather to compensate for declining brain function. Immunotherapy targeting amyloid or Tau has not been an ultimate solution for AD. In addition to SPs and NFTs, oxidative stress, mitochondrial dysfunction, hormone dysregulation, inflammation, mitotic dysfunction, calcium imbalance, and genetic risk factors are all involved in AD processes [9]. The disease is now recognized as multifactorial and consequently strongly demands more effective treatments. Recently, mounting evidence has shown that successful treatment of neurodegenerative diseases, including AD, Parkinson’s disease, and amyotrophic lateral sclerosis, can be achieved through the use of stem cells [288,289,290,291,292,293,294]. A search for the terms “Alzheimer’s disease” and “Stem cells” yields more than 1000 articles in PubMed. Cell therapy may offer an opportunity to treat AD or delay its progression by being able to tackle several factors involved in its pathogenesis at once.

### 8.1. Mesenchymal Stem Cells

Mesenchymal stem cells (MSCs) are widely used for cell therapy because of their easy availability, their ready expansion *in vitro*, the lack of ethical constraints compared to those concerning embryonic stem cells, and their potential use as an autologous transplant that avoids graft rejection and/or side-effects associated with immunosuppression. MSCs can be isolated from a varied range of tissues, such as bone marrow (BM), umbilical cord blood (UCB), adipose tissue, placenta, *etc.* [295,296,297]. In brain disorders, drug delivery is required to go through the BBB; MSCs can cross the BBB and home in on areas of damage. When chemokine receptor type 4 (CXCR4), which reacts to the signaling factor stromal cell-derived factor-1 (SDF-1), is increased in MSCs, homing functions are accelerated for lesioned areas [298]. Although MSCs can migrate to inflammatory sites after intravenous injection, most of the transplanted MSCs might be trapped in the lung instead of reaching lesioned sites with inflammation [299]. In addition to intravascular delivery (vein and artery), different routes have been used to implant MSCs, including direct injection into damaged or lesioned tissue (e.g., intracerebral), intraventricular or intrathecal injection, as well as intranasal application [300,301].

Their paracrine effects, including the production of growth factors and anti-inflammatory cytokines and anti-apoptotic regulation, are strongly exerted and induce neural regeneration, remyelination and immunomodulation [302]. MSCs can reportedly reduce Aβ levels by affecting amyloidogenesis and/or through microglia. Placenta-derived MSCs decreased the expression of APP and BACE1 and the activity of γ-secretase resulting in a significant reduction of Aβ deposition and the improvement of cognitive function [303]. BM-MSCs can increase the population of activated microglia and reduce amyloid deposits through Aβ clearance by phagocytosis [304]. However, microglia secrete high levels of proinflammatory cytokines *in vitro*, such as IL-1β, TNF-α, and IL-6, when stimulated with Aβ [305]. The expression of IL-1β and TNF-α were significantly increased in 9-month-old APP/PS1 mice, but BM-MSC treatment markedly decreased the expression of both cytokines [306]. Aβ toxicity was also reduced by increasing the expression of the anti-inflammatory cytokine IL-4 after MSC treatment. IL-4 is involved in the downregulation of TNF-α and the upregulation of IGF-1 from microglia and also alters the phenotype of Aβ-committed microglia [307,308]. MSCs can produce prostaglandin E2, which modulates inflammatory reactions via the EP2 and EP4 receptors, and can reprogram macrophages to produce more IL-10 [309,310,311]. This anti-inflammatory cytokine, produced by monocytes and macrophages, seems to prevent the migration of neutrophils and reduce oxidative damage [312]. MSCs are likely to exert phagocytic effects on Aβ as well as an anti-inflammatory influence on AD brains via microglia. However, the specific time point at which to apply MSCs needs to be clarified because the conditions in AD brains differ from one stage of AD to the next.

MSCs secrete neurotrophic factors such as vascular endothelial growth factor (VEGF), brain-derived neurotrophic factor (BDNF) and IGF-1 and foster the secretion of BDNF, nerve growth factor (NGF), VEGF and fibroblast growth factor (FGF) 2 in host brain tissues, which may induce endogenous neurogenesis, angiogenesis and neuronal protection [290,312]. Transplantation of MSCs into the subventricular zone (SVZ) or dentate gyrus (DG) has been shown to stimulate the proliferation, differentiation and maturation of endogenous neural stem cells (NSCs) toward a neuronal phenotype [313,314]. Intracerebrally or intravenously injected human adipose-derived MSCs drastically elevated endogenous neurogenesis as well as synaptic and dendritic stability [315]. MSCs transplanted into the lateral ventricle migrated into the hippocampus, including the DG, and enhanced hippocampal neurogenesis [316]. Thus, the interaction between grafted MSCs and endogenous NSCs is crucial for attenuating the neuronal damage and loss observed in AD. In addition, MSCs might be able to protect AD brains from glutamate excitatory-induced apoptosis by secreting growth factors, activating the PI3K/Akt pathway, increasing anti-apoptotic factors and reducing caspase-3 activity [317].

Inhibitory effects of MSCs on Tau pathology have been reported. The intrahippcampal implantation of MSCs significantly reduced hyperphosphorylated Tau, which was suggested to be due in part to a reduction of Aβ_42_ levels [304]; the APP/PS1 mouse model was used for this study. Further studies are needed to elucidate the mechanisms underlying the inhibitory role of MSCs.

### 8.2. Neural Stem Cells and Neurogenesis

Adult neural stem cells (NSCs) are present in the SVZ of the lateral ventricle and the subgranular zone (SGZ) of the hippocampal DG. In the rodent SVZ, more than 30,000 neuroblasts migrate to the olfactory bulb through the rostral migratory stream each day, where they differentiate into granule and periglomerular neurons [318,319]. Young adult rats newly generate approximately 9000 cells in the SGZ every day (*i.e.*, about 6% of total granule cells are generated in the DG each month), but most of these cells die between 1 and 2 weeks after birth [320]. Newly generated neurons from NSCs in the DG are restricted to the formation of mostly DG cells [321].

In aged rodents, the number of NSCs was reduced by 49% in the SVZ, but did not decrease in the SGZ [322]. In addition, Wnt-mediated signaling of astrocytes was reduced with age in the DG, leading to a downregulation of survivin (a mitotic regulator) expression in NSCs and resulting in the quiescence of NSCs in the aged brain [323] and a consequential age-related decline in neurogenesis [324]. NSCs obtained from aged brains are incapable of continuous proliferation and transdifferentiation into neurons because of their shorter telomeres and the lack of telomerase activity [325]. The existence of a quiescent stem cell population in the brain provides a therapeutic opportunity to restore damaged neurons following brain injury and disease.

NSCs are self-renewing and generate multiple neural lineages; after transplantation, NSCs can differentiate into neurons, astrocytes, and oligodendrocytes [326]. In APP knockout mice, transplanted NSCs cannot migrate or effectively differentiate into neurons in the cerebral cortex, since APP secretion from dying cells causes gliogenesis. A damaged APP system may jeopardize normal brain function, and its alteration may lead to excessive gliogenesis [327]. Once a hostile microenvironment is established in AD brains, transplanted NSCs are unlikely to differentiate into mature neurons without proper conditioning against the hostile niche [328]. Neural progenitor cells (NPCs) generated from the adult hippocampus predominantly differentiate into astrocytes, but NPCs transplanted with MSCs into hippocampal slice cultures favored oligodendrogenesis; the MSCs provide a pro-oligodendrogenic microenvironment for the transplanted NPCs [329]. Expression of the neuroprotective gene seladin-1 is decreased in NSCs of the AD brain. These cells are more predisposed to oxidative stress and cell death and might be protected by human BM-MSCs, in which high levels of seladin-1 have been found [328].

### 8.3. Genetically Modified Cells

Advancements in genetic technology enable the introduction or elimination of specific genes in stem cells. Genetically modified cells may have a powerful therapeutic potential to treat AD patients. Toll-like receptors (TLRs) play an important role in the activation of phagocytes/microglia in response to pathogens and damaged host cells in order to clear pathogens, damaged tissue and accumulated waste. Microglial activation by Aβ requires TLR2, TLR4 and TLR6 [330]. CD14 acts as a co-receptor for TLR2 and TLR4, and is required for microglial phagocytosis of Aβ [331]. Aside from TLR3, all TLRs use myeloid differentiation primary response protein 88 (MyD88) as an adaptor [332], which mediates pathogen recognition signaling in immune cells. Aβ deposits are recognized by TLRs and induce inflammatory responses through the MyD88 signaling pathway, resulting in the exacerbation of β-amyloidosis [332]. BM cells genetically modified by deleting MyD88 increase the phagocytic activity of BM-derived macrophages and decrease brain inflammation [333].

NGF prevents neuronal death and improves spatial memory in animal models of aging [334]. However, it cannot be delivered into the CNS via peripheral administration due to its inability to cross the BBB because of its size and polarity [335]. In order to overcome this difficulty, genetically modified cells have been used to ameliorate side effects, including pain and weight loss, [336] and to protect basal forebrain cholinergic neurons. The results of a phase I trial suggested an improvement in cognitive decline [337]. The potential of NGF delivery via a viral vector is under study in an ongoing clinical trial [338].

BDNF is produced in the entorhinal cortex throughout life and is involved in neural plasticity [339]. The level of BDNF declines in the entorhinal cortex and the hippocampus in AD [340]. In 3xTg-AD mice treated with BDNF-secreting NSCs, hippocampal neural density increased and cognition improved without altering Aβ or Tau pathology [326]. On the other hand, in the same transgenic mice, Aβ plaques were reduced in the hippocampus by an intrahippocampal injection of genetically modified NSCs secreting the Aβ-degrading enzyme NEP, resulting in an increase of synaptic density. Non-genetically modified NSCs had no effect on the Aβ plaques [341].

### 8.4. iPS Cells as AD Models

Since Yamanaka and his colleagues introduced induced pluripotent stem cells (iPS cells) in 2006 [342], a new area of stem cell research has been opened. The discovery of iPS cells made possible the development of different types of cellular models of degenerative diseases, including AD. The iPS cell-based AD models offer novel possibilities for deciphering the conundrum of senescent-related pathogenesis. Although they have been successfully generated from cells of a centenarian individual [343,344] and individuals with FAD [345] and SAD [346], they may reset the aging phenotype [347]. Telomere shortening is associated with increasing age to limit the proliferative capacity of stem cells [348]. The telomeres of iPS cells from old donors were elongated similarly as those from young donors [349]. Telomere length and function highly correlate with the pluripotency of iPS cells [350]. In iPS cells generated from the fibroblasts of FAD patients with mutations in PS1 (A246E) and PS2 (N141I), the ratio of Aβ42 to Aβ40 was significantly increased; this increased ratio was reversed by γ-secretase inhibitors [345]. In contrast, iPS cells generated from the fibroblasts of an individual with APP mutations and from the fibroblasts of SAD patients showed significantly high levels of Aβ40, Tau phosphorylation at Thr 231 and active GSK-3β, while the levels of phosphorylated Tau and active GSK-3β were reduced by β-secretase inhibitors, but not by γ-secretase inhibitors [346]. Although these iPS cell models of AD are useful in elucidating the molecular mechanisms of AD pathogenesis without the necessity of obtaining live neurons from AD patients, further studies are required to use iPS cells as a source for AD modeling and treatment.

## 9. Conclusions

The main challenges faced when developing AD treatment include a lack of good animal models that can fully replicate the disease process and symptoms, especially those seen in SAD, as well as a lack of good specific biomarkers to detect and trace AD progression. Current animal models of AD have been mainly generated from ADAD genes that facilitate the AD process. Therefore, the pathological changes and memory deficits typical of AD can be observed at a younger age. However, age is an important risk factor for AD, especially in late-onset AD (SAD), which is much more prevalent among AD patients than early-onset AD. On the other hand, the formation and accumulation of Aβ and Tau, including their oligomers, as well as ER stress, PrP^C^, *O*-GlcNAcylation, oxidative stress, insulin/IGF resistance and glial malfunction are all involved in AD development, and all of them are directly and/or indirectly related to each other in AD pathogenesis and advancement, thereby creating a vicious cycle of AD progression in the brain. Senescence reinforces chronic inflammation including up-regulated TNF-α, IL-1β and IL-6, while oxidative stress is characterized by increased ROS [351], which are also involved in AD pathogenesis [352]. Thus, there are multiple relationships between age-related and disease-related processes. The role of Aβ and hyperphosphorylated Tau, which are both prominent in human AD brains at postmortem autopsy, should be understood in light of senescence-associated molecular mechanisms. Numerous signaling pathways are involved in causing amyloid plaques and hyperphosphorylated Tau. Therefore, to promote our understanding of AD pathogenesis, it might be helpful to consider the AD process in the following three ways: (1) if AD patients have some of the AD-linked genes, the disease will progress following the gene-specific signaling pathways; (2) if some of the metabolic changes advance independently from or without AD-linked genes, the disease will develop in accordance with dysregulated metabolism-dependent signaling pathways; and (3) if genetic factors and early metabolic failure are not involved, metabolic alteration will occur with aging and senescence-induced activation and/or impairment of signaling pathways, resulting in the development of AD. Genetic factors may foster this senescence-dependent AD progression.

Furthermore, a mono-therapeutic approach to AD is not a sufficient way to foster functional improvement in the brain and reverse disease development. AD could be treated according to the cause of the disease at an early stage, but once AD progresses, it would be difficult to interrupt the underlying vicious signaling circuits. Increased or decreased levels of AD-related ligands depend on age, the stage of AD, and the brain region under observation (in which sensitivity to Aβ differs). The systemic application of a reagent targeted to a specific ligand or receptor may exert its effects equally on the ligand distributed throughout the whole brain, where levels of the targeted ligand vary as a result of age and the stage of the disease. Cell therapy can exert a multimodal effect on this multifactorial disease. The beneficial effects of paracrine mechanisms that reduce the overproduction of pro-inflammatory cytokines and induce immunomodulation and multilineage differentiation (or conditioned specific differentiation), which is also done by the transplanted cells themselves, are considered to be very useful for AD treatment. Transplanted cells have the capability to produce and secrete substances into the host tissue. These cells can also be engineered to deliver substances which, in part, activate a population of quiescent NSCs in the SGZ and SVZ, ameliorate the hostile niche created by the vicious cycle of AD and prevent cell apoptosis. Other combinatory therapeutic efforts may be required to correct the AD microenvironment in addition to cell therapy. We must wait for further evidence to answer these key questions: Which cell types are useful in treating or even preventing AD, when is the optimal time period for starting cell therapy, which stages of AD are treatable, how many cells are needed, how often should the AD patient receive treatment, which routes of administration are most suitable for treatment, and so on. Nonetheless, to cut off the development of the vicious AD cycle, our efforts in hunting for the causative culprits of AD among a tangle of many factors must continue.

## Supplementary Materials

Supplementary materials can be found at http://www.mdpi.com/1422-0067/16/11/25961/s1.
