# Supplementary material for: Alzheimer’s Disease: Mechanism and Approach to Cell Therapy
Source: Int J Mol Sci. 2015 Nov 4;16(11):26417–51. doi: 10.3390/ijms161125961 (PMC4661820; doi:10.3390/ijms161125961)
Supplement: Supplementary file 1 [file ijms-16-25961-s001.pdf]

International Journal of
Molecular Sciences

MDPI

Supplementary Information

Materials and Methods

Animals

C57BL/6 male mice (*n* = 25), 129S2/SvHsd male mice (*n* = 24) (The AnLab Ltd., Prague, Czech Republic), and male mice of the triple transgenic mouse model of Alzheimer's Disease (3xTg-AD), which harbour three transgenes of PS1 (M146V), tau (P301L) and APP (SWE) (*n* = 28) (The Jackson Laboratory, Bar Harbour, Ellsworth, ME, USA), were bred in our animal facility. All experiments were performed in accordance with the European Union Directive 2010/63/EU regarding the use of animals in research and were approved by the Ethics Committee of the Institute of Experimental Medicine, Academy of Sciences of the Czech Republic.

Behaviour test

Spatial reference memory was evaluated using a conventional Morris water maze [353,354]. Mice were trained in a circular pool (1.2 m in diameter) that was filled with water made opaque by nontoxic white paint and maintained at 25 °C. An escape platform (10 cm in diameter) was positioned in the middle of one of the quadrants and submerged 1 cm under the water surface. Mice were given 4 trials per day, each of which was started from a different location within each of the quadrants, and trained for 10 days. A probe trial, in which the hidden platform was removed from the pool, was performed 24 h after the last acquisition trial. The latency to find a hidden platform, swimming distance, swimming velocity, and visit frequency to each quadrant were automatically monitored with a video tracking system (VideoMot2, TSE systems GmbH, Bad Homburg, Germany).

Statistical analysis

The data are expressed as mean ± SEM. The comparison of three groups was assessed by a one-way analysis of variance (ANOVA) followed by Turkey's *post-hoc* test. A value of $p < 0.05$ was considered statistically significant.
